# Supplementary material for: Unsupervised topological learning approach of crystal nucleation
Source: Sci Rep. 2022 Feb 24;12:3195. doi: 10.1038/s41598-022-06963-5 (PMC8873400; doi:10.1038/s41598-022-06963-5)
Supplement: Supplementary file 1 — Supplementary Information. [file 41598_2022_6963_MOESM1_ESM.pdf]

Supplementary Information File

Unsupervised topological learning approach of crystal nucleation

Sébastien Becker,[1,2] Emilie Devijver,[2] Rémi Molinier,[3] and Noël Jakse[1]

[1] *Université Grenoble Alpes, CNRS, Grenoble INP, SIMaP*

F-38000 Grenoble, France

[2] *Université Grenoble Alpes, CNRS, Grenoble INP, LIG*

F-38000 Grenoble, France

[3] *Université Grenoble Alpes, CNRS, IF*

F-38000 Grenoble, France

Additional information are presented here to support data and figures of the main text.

SUPPLEMENTARY INFORMATIONS ON THE METHODOLOGY

Properties of the used potentials

Table SI presents some characteristic properties related to each system described by potentials that are used (Ta [1], Al [2] and Mg [3]) in the present classical molecular dynamics simulations. Namely: the melting temperature T_m; the glass transition temperature T_g which were extracted from the quenching of the liquid at ambient pressure with a cooling rate Q up to the amorphous state; the isotherm T_{iso} along which the nucleation process was studied; the ratios $T_{rg} = T_g/T_m$ and $\Delta T = (T_m - T)/T_m$; the estimated critical size of the nuclei n_c; and the critical cooling rate Q_c, up to which the crystallization can be avoided, inferred from the nose of the TTT curves.

	T_m (K)	T_g (K)	T_{iso} (K)	T_{rg}	ΔT	n_c	Q (K/s)	Q_c (K/s)
Ta	3290^a	1582	1900	0.48	0.42	140-150	10^{12}	4.2×10^{11}
Al	926^b	291	650	0.31	0.30	400-800	10^{13}	1.8×10^{12}
Mg	918^c	303	600	0.33	0.35	310-350	10^{12}	3.2×10^{11}

TABLE SI. Characteristic features of the classical molecular dynamic potentials as described in the text. Melting temperatures T_m are taken from Refs. ^{a}[1]; ^{b}[2]; ^{c}[3]

Clustering using a Gaussian mixture Model (GMM)

Figures S1 and S2 show the clustering with the TDA-GMM method applied respectively to Al and Mg configuration during nucleation. The resulting local atomic structures assigned to each cluster are shown. The number of clusters is determined using the ICL criterion. The clustering is performed with Python package `scikit-learn` [5].

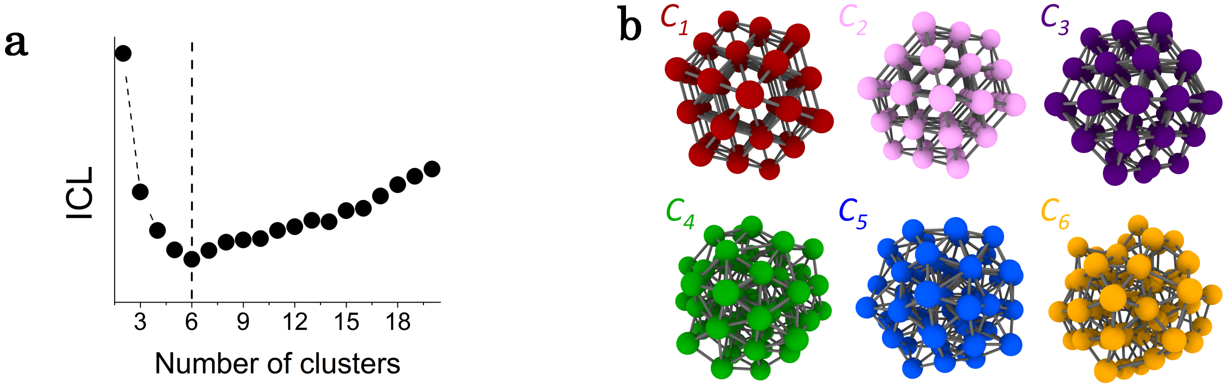

FIG. S1. TDA-GMM clustering of Al. (a) Evolution of the Integrated Completed Likelihood (ICL) criterion as a function of number of clusters. (b) Independent local atomic structures within a cut-off-radius of 6.3 Å form a train set represented in the descriptor space by 173 PH components up to the second order.

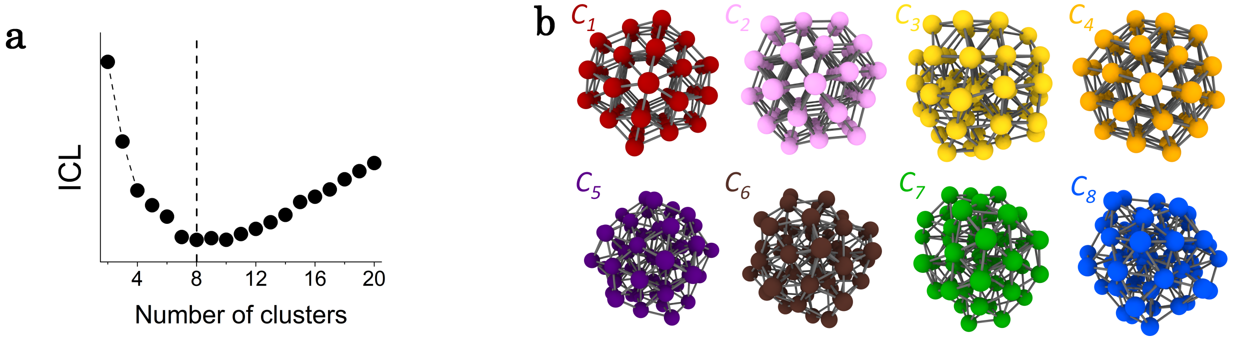

FIG. S2. TDA-GMM clustering of Mg. (a) Evolution of the Integrated Completed Likelihood (ICL) criterion as a function of number of clusters. (b) Independent local atomic structures within a cut-off-radius of 6.9 Å form a train set represented in the descriptor space by 199 PH components up to the second order.

Increasing manually the number of clusters to be treated above the one given by the ICL shows that additional clusters having intermediate orderings thus display a "more continuous" evolution, as can be seen in Figure S3(a). In Fig S3(b), the Principal Component Analysis (PCA) representation [6] of the GMM clustering with 12 clusters over 2 components is given.

We remark that the first axis (which describes 62% of the variance) is describing the crystalline order: most on the right, more crystalline, with the granularity between C_1, C_2, C_3 and the others; whereas the second axis, which explains 8% of the variance, is more about the liquid, with granularity between C_3, C_4, C_5 and C_6. The cluster C_3 has a variation in those two axes. Even if this representation shows a continuum between clusters, we argue that it represents only 70% of the variance, and the remaining one gives differences between the clusters, as they are distinct as given by the posterior probability.

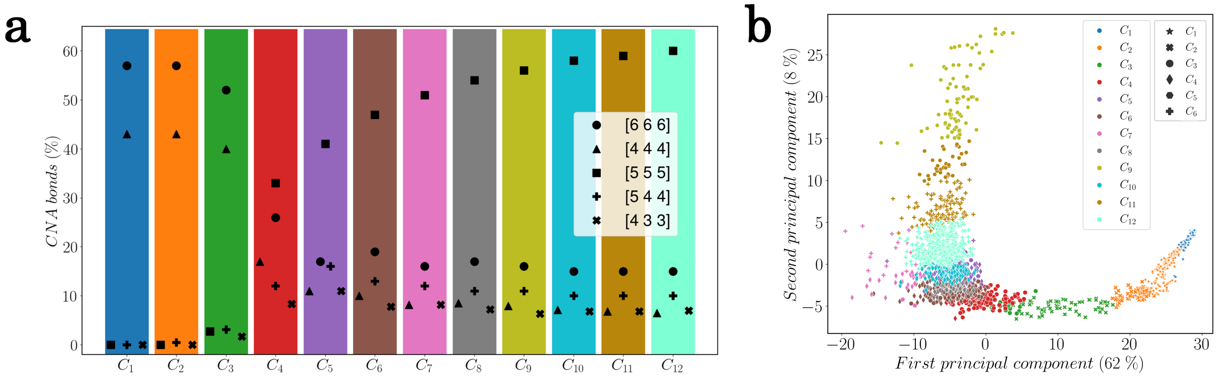

FIG. S3. (a) TDA-GMM clustering of Ta using 12 clusters. (b) Corresponding Principal Component Analysis. Colors are used to distinguish the 12 clusters, while symbols represent clusters to which they belong in the TDA-GMM with 6 clusters given by the ICL criterion.

CRYSTAL NUCLEATION

Identification and extraction of structures during nucleation

Tables SII, SIII and SIV show respectively the evolution of proportion of the structures assigned to each clusters previously identified by the TDA-GMM method in Ta, Al and Mg. As can be seen in each case, clusters C_1 and C_2 follow a fast growth of their proportion along the nucleation process until they are the majority in the final bulk solid. Referring to Figure 1 in the main text and the previous Figures S1 and S2, these two clusters are indeed represented by local atomic structures of pure and distorted crystalline structures. In the

case of Mg, one can notice that clusters C_3 and C_5 are also growing along C_1 and C_2. This is explained by the fact that part of the structures assigned to these two clusters shares bonds with the growing nuclei and carry partial crystalline structure owing to the use of a cut-off corresponding to the second neighbour shell.

Time (ns)	2.70	2.80	2.90	$2.96^{(M)}$	3.00	$3.60^{(S)}$
C_1 (%)	0.09	1.03	5.18	13.44	15.84	83.52
C_2 (%)	0.06	0.33	1.25	4.81	3.27	12.25
C_3 (%)	4.13	4.10	4.12	5.61	4.18	2.92
C_4 (%)	58.11	57.10	53.72	45.56	45.88	0.85
C_5 (%)	3.39	3.38	3.25	3.05	2.84	0.20
C_6 (%)	34.23	34.06	32.48	27.53	28.00	0.27

TABLE SII. Proportion of each cluster for Ta at different times during the nucleation process. Superscripts (M) and (S) correspond respectively to the configuration used to train the model in the TDA-GMM method and the solidified configuration.

Time (ps)	166	170	174	$175^{(M)}$	177	$240^{(S)}$
C_1 (%)	0.05	0.54	3.42	4.24	10.07	36.55
C_2 (%)	0.73	2.29	7.14	9.10	14.34	22.43
C_3 (%)	19.78	19.42	19.74	19.7	20.29	23.57
C_4 (%)	33.22	32.46	29.32	28.85	23.76	9.95
C_5 (%)	44.38	43.53	38.94	36.92	30.60	7.48
C_6 (%)	1.84	1.76	1.43	1.19	0.95	0.02

TABLE SIII. Proportion of each cluster for Al at different times of the nucleation process. Superscripts (M) and (S) correspond respectively to the configuration used to train the model in the TDA-GMM method and the solidified configuration.

Time (ps)	940	960	980	$990^{(M)}$	1000	$1500^{(S)}$
C_1 (%)	0.01	0.13	0.74	3.82	4.60	20.59
C_2 (%)	0.11	0.34	1.32	3.64	5.28	14.58
C_3 (%)	0.31	1.13	3.00	3.98	6.39	12.25
C_4 (%)	0.20	1.37	4.38	3.64	2.38	1.13
C_5 (%)	4.14	5.03	6.68	8.56	10.56	18.30
C_6 (%)	23.91	23.15	21.59	20.78	20.68	19.32
C_7 (%)	36.64	35.30	32.12	28.56	25.30	5.16
C_8 (%)	34.68	33.56	30.17	27.01	24.81	8.68

TABLE SIV. Proportion of each cluster for Mg at different times of the nucleation process. Superscripts (M) and (S) correspond respectively to the configuration used to train the model in the TDA-GMM method and the solidified configuration.

Figure S4 shows the evolution in Mg of the central particles assigned to C_1 and C_2 through the nucleation process along with the TTT curve.

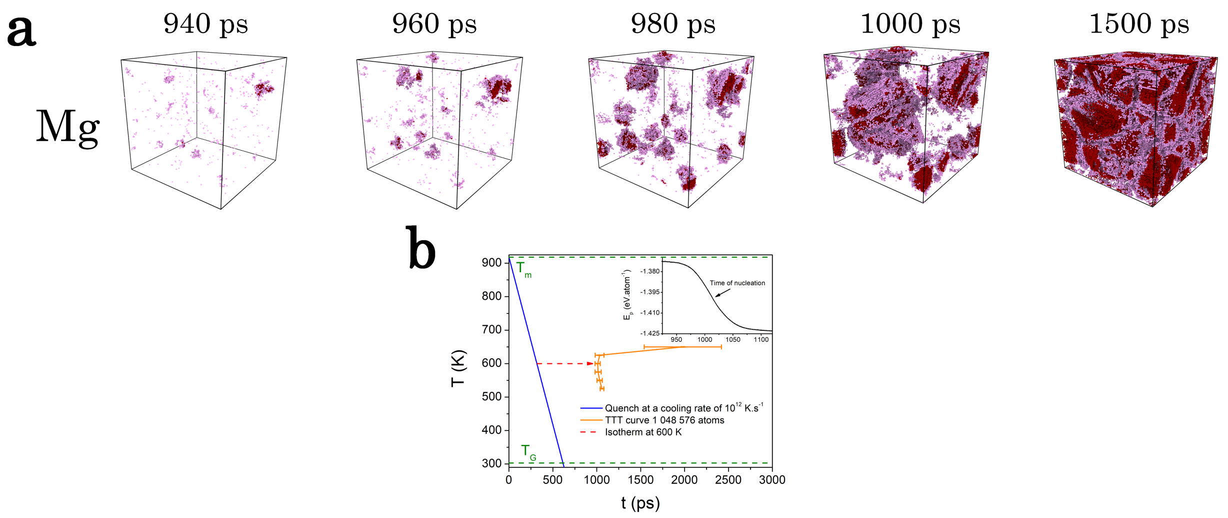

FIG. S4. Homogeneous nucleation events in undercooled Mg during an isothermal process (a) at the nose of the TTT curve (b).

Translational and orientational orderings

Following the procedure described in the main text, a general behaviour for the translational and orientational orderings of Al and Mg is depicted on the Figures S5 and S6. All the nuclei are driven by a concurrent emergence of this two symmetries which correspond respectively to the density of the crystal bulk and the geometrical bonds related to the crystalline local structure.

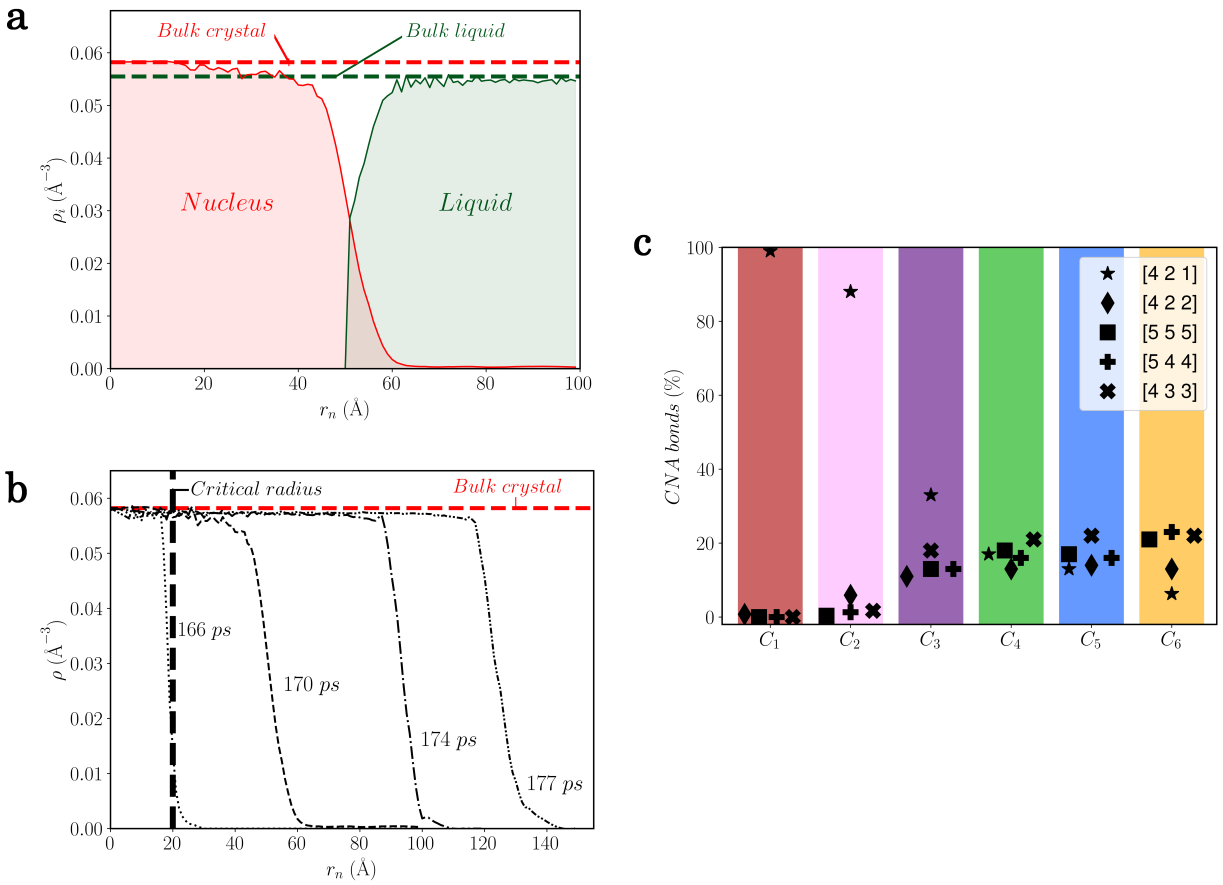

FIG. S5. Typical translational (a) and bond-orientational (c) order parameters for Al. An analysis of the density profile at various times of the biggest growing nuclei (b) shows that the translational order is concurrent with the orientational order at the onset of nucleation.

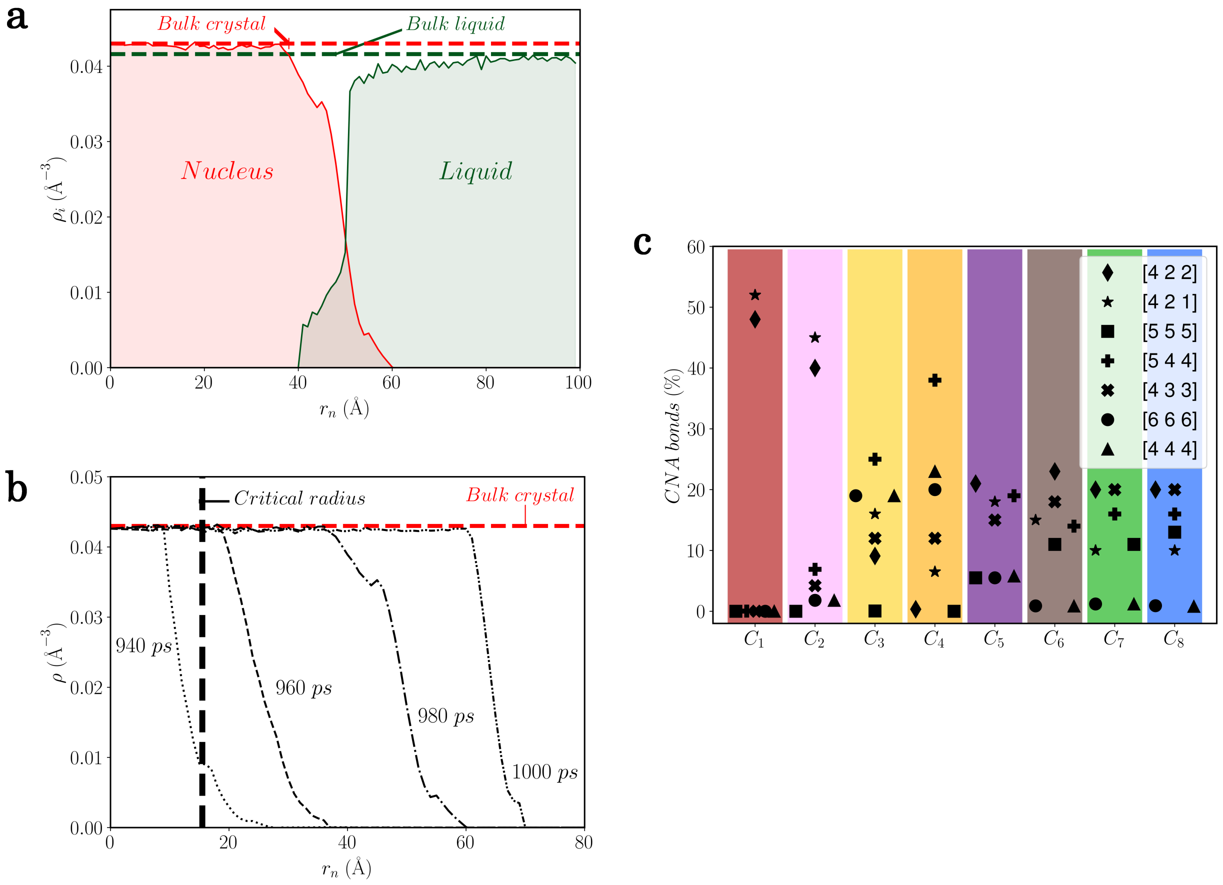

FIG. S6. Typical translational (a) and bond-orientational (c) order parameters for Mg. An analysis of the density profile for various times of the biggest growing nuclei (b) shows that the translational order is concurrent with the orientational order at the onset of nucleation.

BOND-ORIENTATIONAL ORDER ANALYSIS WITH STEINHARDT PARAMETERS

Among the set of parameters based on spherical harmonics which can be computed from the Steinhardt parameters, some of them are widely used in the literature such as the $\bar{q}_2$-$\bar{q}_6$-plane for identification of cristallinity, the $\bar{q}_4$-$\bar{q}_6$-plane for distinction of crystal structure or the $\bar{q}_6$ distribution for identification of solidity [10]. For the Steinhardt descriptors, we used the averaged form of it introduced by Lechner and Dellago [10] by computing all $\bar{q}_i$ order

parameters from $\bar{q}_2$ to $\bar{q}_{12}$ whose expression are

$$\bar{q}_l(i) = \sqrt{\frac{4\pi}{2l+1} \sum_{m=-l}^{l} |\frac{1}{\tilde{N}_b(i)} \sum_{k=0}^{\tilde{N}_b(i)} q_{lm}(k)|^2}$$

in which vector $q_{lm}(i)$ reads

$$q_{lm}(i) = \frac{1}{N_b(i)} \sum_{j=1}^{N_b(i)} Y_{lm}(\mathbf{r}_{ij}),$$

in which $\tilde{N}_b(i)$ is accounting for all the neighbors of a central particle i plus i itself, $N_b(i)$ for the sole number of nearest neighbors and $Y_{lm}(\mathbf{r}_{ij})$ are the spherical harmonics functions of the vector $\mathbf{r}_{ij}$ from particle i to j, with l an integer ranging from 2 to 12 and $m \in [-l, +l]$. Figure S7 shows some of the most informative representations obtained from this parameters on the trained model containing the 6 clusters. The distinction between the structures associated with the liquid and solid clusters is highlighted. Figure **??**(c) is particularly informative on the fact that the structures in C_1 present a well-defined crystalline order, while the distribution in C_2 is more spread out. C_3 shows a boundary distribution between the clusters classified as liquid and the cluster C_2 mainly located at the border of the nuclei. These conclusions are in agreement and equivalent to our results obtained with the CNA. However, although the liquid clusters are identifiable with the Steinhardt parameters, it is not clear that the associated structures also possess a relative crystalline order. On the contrary, the CNA clearly reveals the existence of [666] and [444] bcc geometrical bonds in the structures of these liquid clusters. From these results, there is no reason to think that the use of the CNA itself affects the conclusion concerning the simultaneous emergence of translational and orientational ordering.

Heterogeneity

Heterogeneity at an early nucleation stage is evaluated by a comparison of the distribution of atoms belonging to each cluster against the uniform distribution. If comparison of distributions is well-known in one dimension (using Kolmogorov-Smirnov (KS) test [8]) it is more complex in 3 dimensions as the empirical cumulative distribution function is not

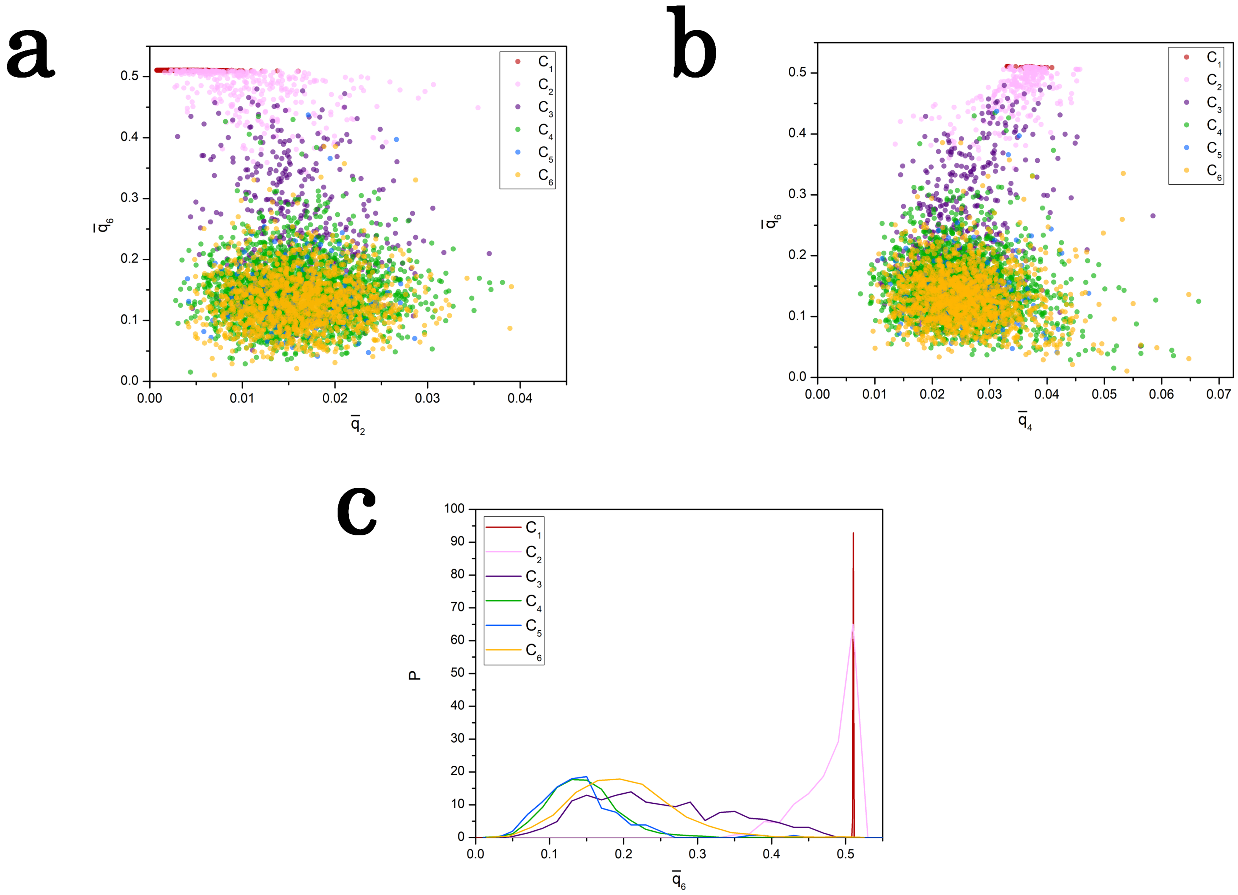

FIG. S7. (a) $\bar{Q}_2$-$\bar{Q}_6$-plane, (b) $\bar{Q}_4$-$\bar{Q}_6$-plane and (c) probability distributions of $\bar{Q}_6$, for the structures in the trained model for Ta.

defined. The following routine is proposed: as soon as the uniform distribution is rejected over a projection, it is rejected for the multivariate dataset; otherwise a nonparametric test named Cramer test based on bootstrap samples and available in the R package `cramer` [9] is used to provide a global conclusion.

The p-values on the 3 axis of the simulation box in Al and Mg in the first configuration at the onset of nucleation (respectively 166 and 940 ps) are depicted on the Tables SV and SVI.

p-value	x	y	z
C_1	0.093	0.000	0.003
C_2	0.000	0.000	0.000
C_3	0.051	0.035	0.398
C_4	0.327	0.197	0.137
C_5	0.525	0.033	0.350
C_6	0.665	0.709	0.898

TABLE SV. p-values computed on the projection of atomic positions at the onset of nucleation in Al on each direction of the simulation box from a KS test against the uniform distribution.

p-value	x	y	z
C_1	0.000	0.000	0.000
C_2	0.000	0.000	0.000
C_3	0.000	0.000	0.000
C_4	0.000	0.000	0.000
C_5	0.000	0.000	0.000
C_6	0.000	0.023	0.000
C_7	0.000	0.000	0.000
C_8	0.017	0.000	0.004

TABLE SVI. p-values computed on the projection of atomic positions at the onset of nucleation in Mg on each direction of the simulation box from a KS test against the uniform distribution.

For Mg, the p-values are particularly low, ensuring the heterogeneity. In the case of Al, the Cramer test for clusters C_3 to C_6 have been used with subsampling of 5000 observations, using 1000 replicates in the bootstrap estimation. The Table SVII shows the p-values obtained with this test. Albeit the uniform hypothesis cannot be rejected here for all the clusters with a level 0.01, one can notice that the p-values still stand more or less far below 1, proving that the distribution of atomic positions is not strictly uniform although it is less obvious that in

the case of Mg or Ta.

Al	p-value
C_3	0.307
C_4	0.112
C_5	0.024
C_6	0.017

TABLE SVII. p-values computed on the atomic positions at the onset of nucleation in Al from a Cramer test in 3D against the uniform distribution.

[1] Zhong, L., Wang, J., Sheng, H., Zhang, Z. & Mao, S. X. Formation of monatomic metallic glasses through ultrafast liquid quenching. Nature 512, 177–180 (2014).

[2] Mendelev, M. I., Kramer, M. J., Becker, C. A. & Asta, M. Analysis of semi-empirical interatomic potentials appropriate for simulation of crystalline and liquid Al and Cu. Philosophical Magazine 88, 1723–1750 (2008).

[3] Wilson, S. R. & Mendelev, M. I. A unified relation for the solid-liquid interface free energy of pure FCC, BCC, and HCP metals. J. Chem. Phys. 144, 144707 (2016).

[4] Becker, S., Devijver, E., Molinier, R. & Jakse, N. Glass-forming ability of elemental zirconium. Phys. Rev. B 102, 104205 (2020).

[5] Pedregosa, F. et al. Scikit-learn: Machine Learning in Python. JMLR 12, 2825 (2011).

[6] Hastie, T., Tibshirani, R., Friedman, J. The Elements of Statistical Learning. New York, NY, USA: Springer New York Inc. (2001).

[7] Stukowski, A. Visualization and analysis of atomistic simulation data with OVITO–the Open Visualization Tool. Modelling Simul. Mater. Sci. Eng. 18, 015012 (2010).

[8] Kolmogoroff, A. Sulla determinazione empirica di una legge di distribuzione. Giornale dell'Istituto Italiano degli Attuari **4**, 83-91 (1933)

[9] Baringhaus, L. & Franz, C., On a new multivariate two-sample test. Journal of Multivariate Analysis, **88**-1, 190-206 (2004).

[10] Lechner, W. & Dellago, C. Accurate determination of crystal structures based on averaged local bond order parameters. The Journal of Chemical Physics 129, 114707 (2008).
